# Phaeohyphomycosis due to *Exophiala* in Aquarium-Housed Lumpfish (*Cyclopterus lumpus*): Clinical Diagnosis and Description

**DOI:** 10.3390/pathogens11121401

**Published:** 2022-11-23

**Authors:** Colin T. McDermott, Charles J. Innis, Akinyi C. Nyaoke, Kathryn A. Tuxbury, Julie M. Cavin, E. Scott Weber, Deana Edmunds, Stéphane Lair, Jill V. Spangenberg, Amy L. Hancock-Ronemus, Catherine A. Hadfield, Leigh A. Clayton, Thomas B. Waltzek, Connie F. Cañete-Gibas, Nathan P. Wiederhold, Salvatore Frasca

**Affiliations:** 1 Department of Veterinary Clinical Sciences, Jockey Club College of Veterinary Medicine and Life Sciences, City University of Hong Kong, Kowloon Tong, Hong Kong 999077, China; 2 Animal Care, New England Aquarium, Boston, MA 02110, USA; 3 California Animal Health and Food Safety Laboratory, San Bernardino Branch, University of California Davis, San Bernardino, CA 92408, USA; 4 Gulfarium Marine Adventure Park, Fort Walton Beach, FL 32548, USA; 5 Department of Medicine and Epidemiology, School of Veterinary Medicine, University of California, Davis, CA 95616, USA; 6 Faculté de Médecine Vétérinaire, Université de Montréal, St. Hyacinthe, QC J2R-J2T, Canada; 7 Aquarium du Québec, Quebec City, QC G1A 0A0, Canada; 8 Aquarium of the Bay, Embarcadero at Beach St., San Francisco, CA 94133, USA; 9 Charles River Laboratories, Wilmington, MA 01887, USA; 10 Seattle Aquarium, Seattle, WA 98101, USA; 11 Animal and Plant Health Inspection Services, U.S. Department of Agriculture, Gainesville, FL 32606, USA; 12 Fungus Testing Laboratory, Department of Pathology and Laboratory Medicine, Long School of Medicine, University of Texas Health San Antonio, San Antonio, TX 78229, USA; 13 Department of Pathobiology and Veterinary Science, University of Connecticut, Storrs, CT 06269, USA

**Keywords:** phaeohyphomycosis, *Exophiala*, *Cyclopterus lumpus*, lumpfish, melanized fungus

## Abstract

Phaeohyphomycosis caused by *Exophiala* species represents an important disease of concern for farmed and aquarium-housed fish. The objective of this study was to summarize the clinical findings and diagnosis of *Exophiala* infections in aquarium-housed *Cyclopterus lumpus*. Clinical records and postmortem pathology reports were reviewed for 15 individuals from 5 public aquaria in the United States and Canada from 2007 to 2015. Fish most commonly presented with cutaneous ulcers and progressive clinical decline despite topical or systemic antifungal therapy. Antemortem fungal culture of cutaneous lesions resulted in colonial growth for 7/12 samples from 8 individuals. Amplification of the internal transcribed spacer region (ITS) of nuclear rDNA identified *Exophiala angulospora* or *Exophiala aquamarina* in four samples from three individuals. Postmortem histopathologic findings were consistent with phaeohyphomycosis, with lesions most commonly found in the integument (11/15), gill (9/15), or kidney (9/15) and evidence of fungal angioinvasion and dissemination. DNA extraction and subsequent ITS sequencing from formalin-fixed paraffin-embedded tissues of seven individuals identified *E. angulospora, E. aquamarina*, or *Cyphellophora* sp. in four individuals. Lesion description, distribution, and *Exophiala* spp. identifications were similar to those reported in farmed *C. lumpus*. Antemortem clinical and diagnostic findings of phaeohyphomycosis attributable to several species of *Exophiala* provide insight on the progression of *Exophiala* infections in lumpfish that may contribute to management of the species in public aquaria and under culture conditions.

## 1. Introduction

Lumpfish *Cyclopterus lumpus* L. 1758 are a semi-pelagic fish, widely distributed in the North Atlantic Ocean with a western range from the U.S.A. to Arctic Canada and an eastern range from the coast of Murmansk, Russia, to the south of Portugal [1,2]. Referred to as lumpsucker, seasnail, henfish, lump, paddle-cock, and poule de mer [3], lumpfish are commercially important in Canada, Greenland, Iceland, and Norway [4], with females targeted for their meat and roe [1,5]. In their most recent commercial application, lumpfish have been used for biological control of sea-lice in salmon farming [6,7]. Modification of their pelvic fins into a ventral sucker allows them to adhere to coastal rock structures, algae, and debris, contributes to their unique body shape, and makes them a popular aquarium exhibit fish [3].

Melanized fungal infections represent an important disease concern in aquaculture because of their high morbidity and mortality with a lack of approved treatment options. A recent retrospective review of aquarium-housed fish submissions to a university pathology service identified melanized fungi in 34 different fish species over a 14-year period. Of the pathogenic melanized fungi, *Exophiala* spp. were the most commonly identified, although culture and molecular identification of fungal species were not available in most cases [8]. Waterborne *Exophiala* spp., order Chaetothyriales, have been known to cause cutaneous and disseminated infections in fish, crabs, amphibians, and turtles. Animals with moist skin are considered to be more susceptible to infection by these filamentous black fungi [9]. Pathogenic *Exophiala* spp. causing infection in cold-blooded animals generally belong to the ‘salmonis clade’ and the ‘*E. angulospora* complex’ [9,10]. *Exophiala salmonis* infections have been reported in Atlantic salmon (*Salmo salar* L.) and smooth dogfish (*Mustelus canis*) [11,12,13], *E. pisciphila* in channel catfish (*Ictalurus punctatus*), Atlantic salmon (*Salmo salar* L.), cardinal tetra (*Paracheirodon axelrodi*), and pretty tetra (*Hemigrammus pulcher*) [14,15,16,17], *E. xenobiotica* in striped jack (*Pseudocaranx dentex*) and Queensland grouper (*Epinephelus lanceolatus*) [18,19], and *E. aquamarina* in weedy (*Phyllopteryx taeniolatus*) and leafy seadragons (*Phycodurus eques*), little tunnyfish (*Euthynnus alletteratus*), winter flounder (*Pseudopleuronectes americanus*), sharphead flyingfish (*Hirundichthys oxycephalus*), and lumpfish (*Cyclopterus lumpus*) [9,10,20]. *Exophiala angulospora* inhabits cold waters worldwide, is considered an opportunistic pathogen with invasive potential and dissemination in cold-blooded vertebrates, and it has been reported in aquarium-housed weedy seadragons (*Phyllopteryx taeniolatus*), Atlantic halibut (*Hippoglossus hippoglossus*), farmed Atlantic cod (*Gadus morhua*), and farmed Japanese flounder (*Paralichthys olivaceus*) [9,10,21,22,23]. Cutaneous infections in humans with *E. salmonis* and *E. pisciphila* have been reported, highlighting potential zoonotic risks [9,24,25]. *Exophiala dermatiditis*, *E. xenobiotica*, and *E oligosperma* have been identified in human infections, ranging in severity from superficial cutaneous infection to systemic mycosis [9,26].

Tissue infection by melanized fungi-forming hyphae is referred to by the general term phaeohyphomycosis and can be superficial or deep. Clinical signs of phaeohyphomycosis in fish are generally non-specific and include weight loss, lethargy, anorexia, listing in water column, abnormal buoyancy, single to multiple skin ulcers, and death with or without premonitory signs [10,14,15,27]. Findings on gross examination include cutaneous ulcers and multiple black foci in viscera and gills. Microscopic lesions consist of necrosis with infiltrates of inflammatory cells centered on slender, variably brown, septate fungal hyphae. Macrophages are the predominant cell in these infiltrates, but lymphocytes and granulocytes may also be present [8]. Depending on the fish species, inflammatory infiltrates may not form granulomas but instead may be loosely organized in the necrotic tissue and admixed with hyphae. A common feature of these infections is fungal angioinvasion with hyphae traversing the endothelium and walls of affected blood vessels and being present in vascular lumina [8,10]. Initial infection is suspected to occur across the gills or skin via inoculation or through preexisting lesions [10]. *Exophiala* spp. are ubiquitous in soil and aquatic environments. Environmental persistence of *Exophiala* spp. has been suggested as the source of infection for cultured and aquarium fish [7,10,23]. Although there is evidence for in vitro efficacy of antifungal medications against *Exophiala* spp. [28], phaeohyphomycosis carries a guarded prognosis in fish [8,29]. This is attributed to a combination of virulence factors in melanized fungi, the progressive nature of the disease, and a general lack of veterinary antifungal options in the treatment of systemic fungal infections [8,9,29]. Treatment options are further restricted in aquaculture due to a lack of approved antifungal agents [30]. Attempted treatment of *E. angulospora* infected *C. lumpus* broodstock with a combination of formalin 200 ppm and bonopol 40 ppm was not effective in resolving fungal infections or in disinfecting the environment [7].

Phaeohyphomycosis caused by *Exophiala* spp. in lumpfish represents an important disease concern in aquaculture. Infections by *Exophiala* spp. in lumpfish have primarily been reported from European fish farms [6,7,31,32]. *Exophiala angulospora* has been reported to cause multifocal chronic inflammatory lesions of the skin, gills, and internal organs of hatchery-raised lumpfish in Scotland [7]. Multiple species of *Exophiala*, including *E. angulospora*, *E. salmonis*, and *E. psychrophila*, and a species of *Cyphellophora* have been described in lumpfish in Ireland [32]. *Exophiala psychrophilia* and a *Cyphellophora* sp. have been described in farmed lumpfish reared for future broodstock [31,32]. In addition to phaeophyphomycosis, several parasitic [33,34,35,36,37,38] and bacterial [34,39,40,41,42] infections have been reported in wild, farmed, and aquarium-housed lumpfish.

Previous reports of melanized fungal infections in lumpfish have focused on postmortem findings in farmed fish. In this retrospective report, we highlight clinical observations, attempted treatment, gross, microscopic, and molecular findings of phaeohyphomycosis in aquarium-housed *C. lumpus* from multiple geographically diverse aquaria in North America, with identification of *E. angulospora, E. aquamarina,* and *Cyphellophora* sp.

## 2. Materials and Methods

### 2.1. Animals

Specimens consisted of 15 adult, captive-bred and wild-collected lumpfish (*Cyclopterus lumpus*) from 5 separate public aquaria in the U.S.A. and Canada. Cases of histologically confirmed phaeohyphomycosis that were documented between 2007 and 2015 were reviewed. Information gathered from medical records included clinical findings, temporal course of the disease, antemortem diagnostic testing (cytology, surgical biopsy, and microbiology), and attempted treatments. The results of postmortem examination, histopathology, microbiology, and molecular testing were reviewed.

In total, there were 9 females, 5 males, and 1 individual whose sex was not indicated in the medical record. Five individuals were definitively identified as wild caught, two individuals were captive bred, and the source was not recorded in the medical record for eight individuals. Weights were recorded at necropsy for 11 individuals: 7 females (average 2.73 kg, range—1.80–3.40 kg) and 4 males (average 0.68 kg, range—0.54–0.87 kg).

Animals were housed in both species-specific and mixed species tanks (2100–44,600 L) in filtered, natural, or artificial seawater at temperatures of 4–12 °C (39.2–53.6 °F), pH 7.6–8.3, and salinity 27–32 g/L.

### 2.2. Antemortem Cytology

When performed, skin cytology was collected from areas of cutaneous ulceration. The surface layer of the skin or ulceration was scraped with a surgical blade, cover slip, or hypodermic needle of appropriate size to release an adequate sample from the tissues and immediately viewed directly under light microscopy as a wet mount preparation.

### 2.3. Antemortem Microbiology

In cases where a tentative identification was made using in-house cytology, tissue samples and culture swabs were submitted to different clinical diagnostic laboratories at the discretion of the attending veterinarians for microbial testing, including separate aerobic bacterial cultures and fungal cultures. For bacterial isolation, representative swabs were plated onto Trypticase Soy Agar with 5% Sheep Blood (Becton Dickinson, Franklin Lakes, NJ, USA) and MacConkey II Agar (Becton Dickinson, Franklin Lakes, NJ, USA) by using standard methods at 25 °C (77 °F) and 35 °C (95 °F) for 18–24 h.

Fungal cultures of the skin or combination skin and muscle were either collected from surgical wedge or punch biopsies under anesthesia, or from superficial swabs of the representative lesions. In both cases, the skin was lightly rinsed with sterile saline or sterile water prior to collection to reduce surface debris prior to sampling.

For fungal culture, swabs were used to streak potato dextrose agar slants and incubated at 22–25 °C (71.6–77 °F). Cultures were monitored for up to three weeks for the growth of fungal colonies. In cases where an olivaceous to black, velvety colony was isolated, a sub-culture was started on a second potato dextrose agar slant.

### 2.4. Necropsy and Histopathology

Individuals were either euthanized due to clinical decline or found dead. Gross necropsies were performed on all individuals. Representative tissue samples or swabs of lesions were collected for wet mount preparation and microbial culture at the discretion of the attending clinician. Multiple tissue samples were fixed by immersion in 10% neutral buffered formalin. 

Lumpfish were submitted to one of four diagnostic pathology laboratories (Connecticut Veterinary Medical Diagnostic Laboratory, Storrs, CT, USA; Department of Molecular and Comparative Pathobiology, Johns Hopkins University, Baltimore, MD, USA; Centre québécois sur la santé des animaux sauvages, Université de Montréal, St Hyacinthe, QC, Canada; California Animal Health & Food Safety Laboratory System, San Bernardino, CA, USA). In brief, formalin-fixed tissue samples were trimmed to fit plastic cassettes, routinely processed, embedded in paraffin, sectioned at 4–6 µM, mounted on glass slides, stained with hematoxylin and eosin or hematoxylin, phloxine and saffron, and then examined by bright-field microscopy. Additional sections were stained with either Fontana–Masson, Grocott’s methenamine silver, or Schmorl’s reduction staining techniques to highlight histomorphologic and staining characteristics of the fungi.

### 2.5. Fungus Identification and Antifungal Drug Susceptibility Testing

In cases of positive fungal cultures, isolates obtained from skin (T12068 = UTHSC 13-1953), tail lesion (3152 = UTHSC 07-871), pectoral fin (9404 = UTHSC 15-2163), and skin wound (T12069 = UTHSC 13-1506 and 3189 = UTHSC 07-1926) were sent to the Fungus Testing Laboratory (FTL), University of Texas Health Science Center (UTHSC) at San Antonio, San Antonio, TX for identification. Cultures were grown on potato flakes agar plates (PFA) for morphologic assessment and molecular procedures. Growth at 30, 37, and 40 °C (86, 98.6, and 104 °F) was also assessed.

Genomic DNA was extracted from culture on PFA. The internal transcribed spacer (ITS) region of the nuclear rDNA was amplified and sequenced following previously described methods [43]. The generated ITS sequences were used to compare with sequences available in GenBank by BLAST search. FTL local BLAST using an updated Barcode identifier database for herpotrichiellaceous black yeasts and relatives [44], and phylogenetic analyses were done to confirm the identity of the isolates. ITS sequences were analyzed together with sequences of representative reference and type strains of closely related taxa using Maximum Likelihood (ML). The ML tree was calculated in IQ-Tree v2.2.0 [45] with the optimal model determined using the Modelfinder [46], and ultrafast bootstrapping was performed using UFBoot2 [47]. The most suitable model was chosen based on the Akaike information criterion. All of these were integrated in IQ-Tree. Bayesian inference was conducted using MrBayes v3.2.7 [48]. The phylogenetic tree was visualized in Figtree v.1.4.4 (http://github.com/rambaut/figtree/ downloaded on 26 August 2019, tree visualized on 11 August 2022) and visually edited in Canvas Draw v.3.0.5 (build 274).

Minimum inhibitory concentrations (MICs) for amphotericin B (AMB), fluconazole (FLU), itraconazole (ITRA), voriconazole (VORI), terbinafine (TRB), and miconazole (MON) were determined by visual observation against four isolates from three fish (3152, 3189, and T12069) by the broth microdilution method as described in the CLSI M38 standard [49]. RPMI-1640 (with glutamine and phenol red, without bicarbonate) buffered to pH 7 with 0.165M 4-morpholinepropanesulfonic acid (MOPS) was the growth medium. The MIC endpoint for amphotericin B, itraconazole, voriconazole, and terbinafine was the lowest concentration that resulted in 100% growth inhibition by visual observation. The MIC endpoint for fluconazole and miconazole was the lowest concentration that resulted in at least 50% growth inhibition compared to the drug-free growth control, as described by the CLSI M38 standard. MICs we read after 48 h of incubation.

### 2.6. DNA Extraction and PCR Detection of Exophiala from Formalin-Fixed Paraffin-Embedded Tissues

Tissue samples were cut from paraffin blocks of formalin-fixed paraffin-embedded (FFPE) lumpfish tissues in 50 µM sections. Each cut used a new microtome blade surface to reduce the risk of cross-contamination. Qiagen deparaffinization solution, ATL buffer, and Proteinase K were then added to samples and incubated at 56 °C (132.8 °F) overnight before extraction of DNA using a DNA FFPE Tissue Kit (Qiagen) according to the manufacturer’s instructions. To screen lumpfish DNA samples for *E. angulospora* and *E. aquamarina*, a primer set was designed to amplify a portion of the ITS1 region based on these two fungi (Table A1). The ITS1 and flanking sequences for *E. angulospora* and *E. aquamarina* were retrieved from GenBank and aligned in MAFFT 7 [50] using default settings, and the resulting alignment was imported into Geneious R10 [51] to generate a consensus sequence with the threshold set to 100%. The consensus sequence was imported into Primer3 (https://primer3.ut.ee/ accessed on 1 April 2015) to design primers with the following characteristics: conserved primer binding sites 150–250 bp apart with a hypervariable region between to facilitate the discrimination of *E. angulospora* and *E. aquamarina* by Sanger sequencing. The designed primers amplified approximately 180 bp of the fungal ITS1. Using the method described above, a second primer set was designed to amplify a 245 bp region of the lumpfish cytochrome oxidase subunit 1 (COX1) gene to serve as a housekeeping genetic target for assessment of the ability of DNA samples to support PCR amplification (Table A1). Separate PCR reactions designed to amplify the lumpfish COX1 and fungal ITS1 genetic targets were used to screen DNA extracts generated from each FFPE lumpfish tissue sample. DNA extracts testing negative using the lumpfish COX1 PCR assay were not tested with the *Exophiala* ITS PCR assay since true-negative results could not be distinguished from false negative results (type II error) that may have occurred because of PCR inhibition and/or DNA degradation, which may have occurred prior to necropsy and processing or as a result of adverse storage conditions of lumpfish tissues before or after embedding in paraffin (e.g., prolonged storage in formalin).

Reaction volumes for both the *Exophiala* ITS1 and lumpfish COX1 PCR assays were 50 µL and consisted of 0.25 µL of Platinum Taq DNA Polymerase (Invitrogen), 5.0 µL of 10× PCR Buffer, 2.0 µL of 50 mM MgCl_2_, 1.0 µL of 10 mM dNTPs, 2.5 µL of 20 µM forward and reverse primers, 32.25 µL of molecular-grade water, and 4.5 µL of DNA template. An initial denaturation step of 94 °C for 5 min was followed by 40 cycles of a 94 °C denaturation step, a 55 °C annealing step, and a 72 °C extension step, with each step run for 30 s, followed by a final extension step at 72 °C for 10 min. PCR products were subjected to electrophoresis in 1% agarose gels stained with ethidium bromide. Amplified products were purified using a QIAquick PCR Purification Kit. The concentration of purified DNA was quantified fluorometrically using a Qubit^®^ 3.0 Fluorometer and dsDNA BR Assay Kit (Life Technologies, Carlsbad, CA, USA). Purified DNA was sequenced in both directions on an ABI 3130 platform (Applied Biosystems, Foster City, CA, USA). The sequence data were assembled and edited in CLC Genomics Workbench 7.5 software (Qiagen, Venlo, The Netherlands). Primer sequences and regions of low-confidence base calling were excluded at both the 5′ and 3′ sequence ends. BLASTN analysis was performed using the edited and assembled sequences (https://blast.ncbi.nlm.nih.gov/ accessed on 1 September 2022).

## 3. Results

### 3.1. Observed Clinical Signs

**Figure 1:**
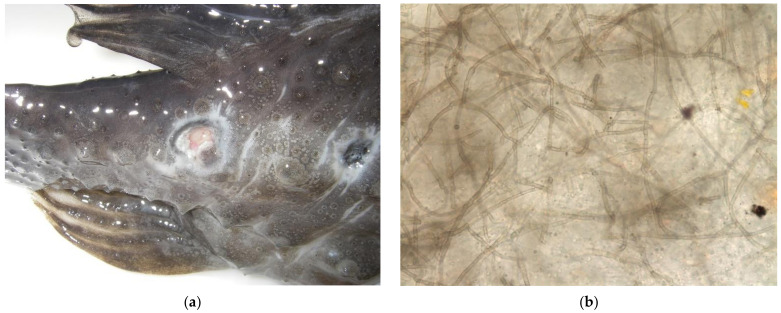

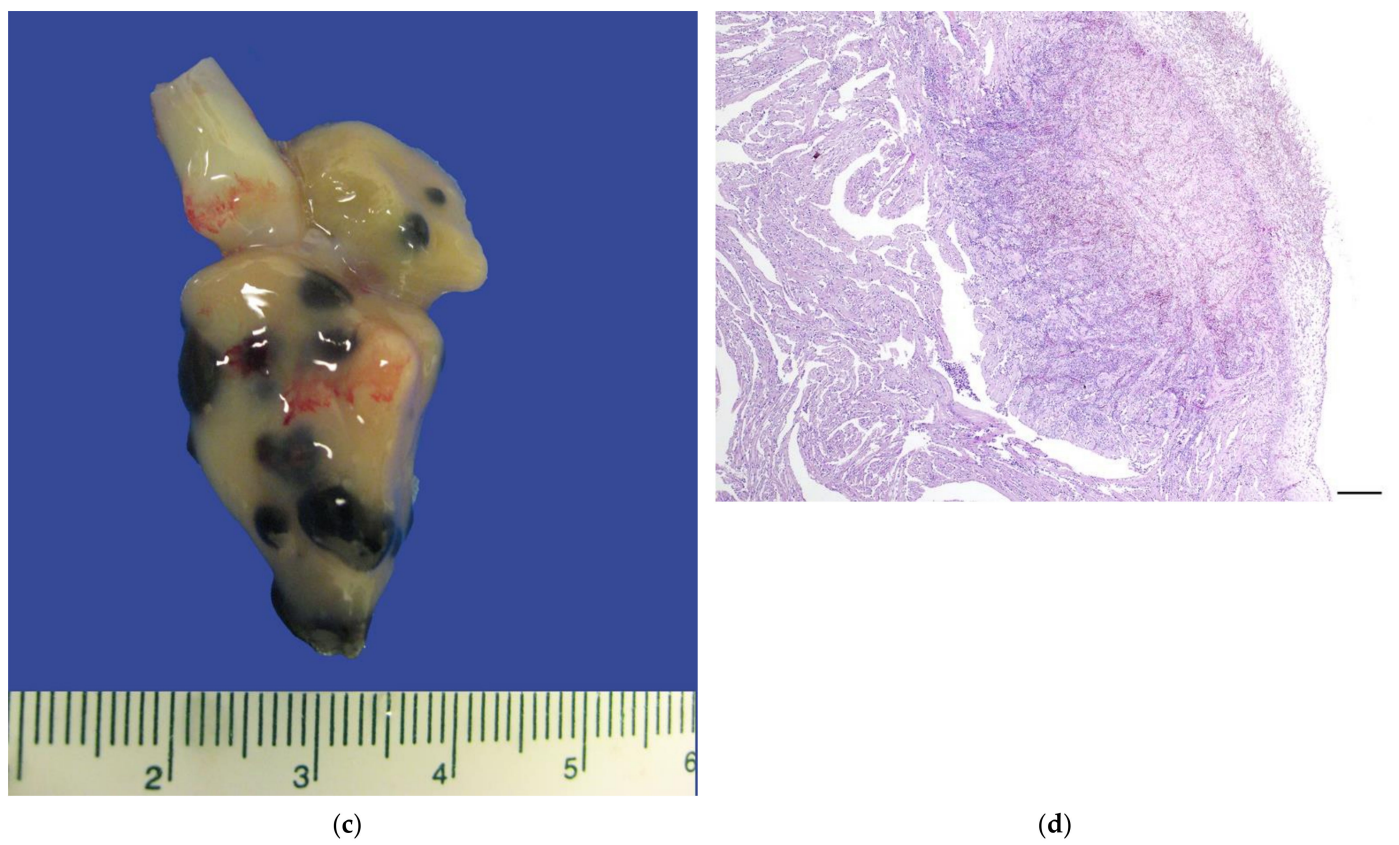
(**a**) Integumentary lesions representative of *Exophiala* infection in lumpfish. Ulcers are well demarcated with raised margins and central depressions. (**b**) Wet mount cytology of a cutaneous ulcer from the lumpfish in (**a**). Fungal hyphae are light brown and slender with parallel walls, transverse septa, and lateral branchings. (**c**) *Exophiala* infection of the heart from a lumpfish. Multiple, discrete, and occasionally coalescent, slightly raised, black foci are scattered in the ventricle and atrium. (**d**) Histologic section of the heart of a lumpfish with black multifocal lesions displayed in (**c**). Melanized fungal hyphae are numerous and form a mat extending from the epicardial surface and invading the underlying myocardium. H&E, bar = 200 µM.

Clinical signs of affected fish included one or more of the following: progressive skin erosions, weakness, loss of appetite, lethargy, increased respiratory rate and effort, abnormal buoyancy, or listing. External lesions consisting of one to several, well-demarcated, extensive, cutaneous ulcers with raised black margins distributed along the body were noted antemortem in 12 cases (Figure 1a). Clinical signs were present prior to death or euthanasia in 12 cases; duration of clinical signs ranged from 1 to 139 days (mean = 42.2 days). No clinical signs were noted prior to death in three individuals. Seven individuals were euthanized due to a lack of clinical response to treatment, and three individuals were found dead while on medical therapy. The definitive method of death was not recorded in two individuals.

### 3.2. Antemortem Diagnostics and Treatments

#### 3.2.1. Diagnostics

Direct wet preparation cytology was performed in 11 cases of 12 external lesions. Of these cases, variably brown fungal hyphae with parallel walls, transverse septa, and lateral branching consistent with a phaeohyphomycete were noted in 10 individuals on antemortem direct wet preparation skin cytology (Figure 1b). In one individual, only necrotic debris and ciliated protozoa were present within an antemortem cytologic sample from the cutaneous lesion. Scuticociliates consistent with *Uronema* spp. were present in six individuals. In four individuals, these ciliates were identified on earlier skin cytologic samples, prior to finding fungal elements. Monogenean parasites consistent with *Dactylogyrus* spp. were identified on skin scrapes in three individuals.

Definitive antemortem diagnosis of cutaneous fungal infection was made via antemortem skin biopsy in three individuals. Twelve antemortem fungal cultures of skin biopsies or swabs of cutaneous lesions were attempted in eight individuals, and seven of those cultures yielded pigmented fungal growth (Table 1).

Antemortem aerobic bacterial cultures were performed in three lumpfish. One antemortem culture was positive for growth of a *Micrococcus* sp. from a skin lesion (T12068). Antemortem blood culture from one individual (6192) and an aspirate of a cutaneous cyst from another individual (6193) were negative for bacterial growth.

#### 3.2.2. Attempted Treatments

After the initial tentative diagnosis of *Exophiala* spp. infection, individuals were treated at the discretion of the attending veterinarian using a variety of antifungal drugs, including itraconazole (2.5–20 mg/kg PO q48hr, 6 individuals), terbinafine (12.5–25 mg/kg PO q24hr or 0.005% bath for 5 min q5d, 3 individuals), methylene blue (topical), miconazole (topical, 2 individuals), amphotericin B (0.8–1 mg/kg intraperitoneal or local injection q48hr, 2 individuals), fluconazole (60 mg/kg PO, 1 individual), and surgical excision of skin lesions (2 individuals). None of these treatments were considered effective in eliminating fungal infections due to the persistence of fungal elements upon histopathologic examination of tissues from all treated individuals. Therapy for concurrent bacterial and parasitic infections varied and included at least one of the following compounds: enrofloxacin (5 mg/kg PO or IM q48hr, 4 individuals), ceftazidime (20–22 mg/kg IM q72hr, 3 individuals), ceftiofur (7 mg/kg IM single dose, 1 individual), 37% *w*/*v* formaldehyde solution (25–250 ppm immersion q72hr, 6 individuals), malachite green (in combination with formalin, 1 cc/10 gallons, 1 individual), vitamin C supplementation (dose not specified in record, 1 individual), praziquantel (5 mg/kg PO once, one individual), and meloxicam (0.2–0.4 mg/kg PO or IM q48–72hr, 5 individuals).

### 3.3. Necropsy and Histopathology

#### 3.3.1. Gross Necropsy

On internal examination of each lumpfish, well-demarcated, and occasionally extensive black foci were identified in one or more of the following tissues: gill, kidney, liver, heart, spleen, intestinal serosa, coelomic membrane, gonad, stomach, and/or choroid of the eye (Figure 1c,d). External lesions of the skin, present in 12 individuals, included one to several well-demarcated, extensive ulcers with raised black margins distributed along the body.

In one individual (6193), an ulceration of the dorsal ridge had the appearance of healing during treatment with oral itraconazole and terbinafine baths. Direct wet preparation cytologic examination of the healing ulceration did not reveal fungal elements.

#### 3.3.2. Histopathology

Microscopically, phaeohyphomycotic lesions were identified in skin (11/15), gill (9/15), kidney (9/15), liver (7/15), heart (6/15), spleen (6/15), intestine (4/15), coelomic membrane (3/15), gonads (2/15), stomach (2/15), eye (1/15), and/or mesentery (1/15) (Table 2). Single lesions were observed in four individuals, and multiple lesions were observed in 11 cases. Lesions were restricted to the skin in three individuals (20% of cases).

Cutaneous lesions were characterized by epidermal ulceration, in some cases extending to the dermal bone and cartilage, with high numbers of intertwined brown fungal hyphae accompanied by varying amounts of protein-rich edema, fibrin, necrotic cells, and karyorrhectic debris.

**Figure 2:**
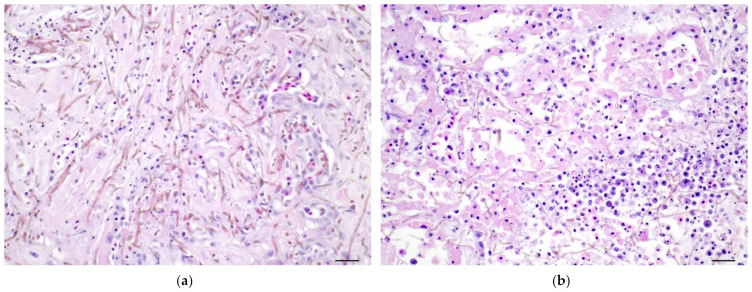
(**a**) Melanized (brown) fungal hyphae are present throughout a focus of the ventricular myocardium wherein many myocardial fibers are necrotic, represented by pale eosinophilic sarcoplasm lacking striations and nuclei or having pyknotic nuclei. (**b**) Melanized (brown) fungal hyphae course through the renal interstitium with many necrotic hematopoietic cells and tubules lined by necrotic epithelial cells having eosinophilic cytoplasm and pyknotic nuclei. H&E, bar = 20 µM.

Branchial lesions consisted of fungal hyphae infiltrating the epithelium and in some cases the cartilage and bone of the gill ray, with infiltrates of macrophages, lymphocytes, and granulocytes. Lesions in the heart consisted of regions of myocardial fiber necrosis infiltrated by numerous fungal hyphae and a mix of inflammatory cells (Figure 2a). Renal lesions included multiple areas of necrosis of tubules and hematopoietic interstitium associated with invasion by fungal hyphae (Figure 2b). Fungal hyphae could be found within the lumen of blood vessels, within the lumina of tubules, and in capsular connective tissue.

Liver lesions ranged in severity from discrete granulomas to multifocal and extensive vascular and hepatocytic necrosis. Fungal hyphae could be found within areas of necrosis or throughout the hepatic parenchyma and vasculature.

Lesions in the spleen included hyperplasia and hypertrophy of the mesothelium with edema, fibrin, hemorrhage, and infiltrates of moderate numbers of viable and necrotic macrophages along the capsule and adjacent mesentery, indicative of capsulitis and mesenteritis. Melanized fungal hyphae were present in the capsular inflammatory infiltrate and subjacent splenic parenchyma. Lesions in gonads were found in the testes of one individual with focal necrosis and infiltration of fungal hyphae. Lesions of the gastrointestinal tract occurred in the stomach and in the serosa and submucosa of the intestine. In the stomach, there was extensive transmural necrosis with edema, hemorrhage, necrotic debris, infiltrates of macrophages and lesser numbers of lymphocytes. In the intestine of one individual, there was focal transmural necrosis accompanied by histiocytic infiltrates, fibrin, serum protein, and high numbers of melanized fungal hyphae throughout the necrotic focus and into the surrounding mesentery. In one individual, a segment of choroid contained high numbers of melanized fungal hyphae with infiltrates of medium numbers of macrophages, granulocytes, necrotic cells, cellular debris, and edema.

**Figure 3:**
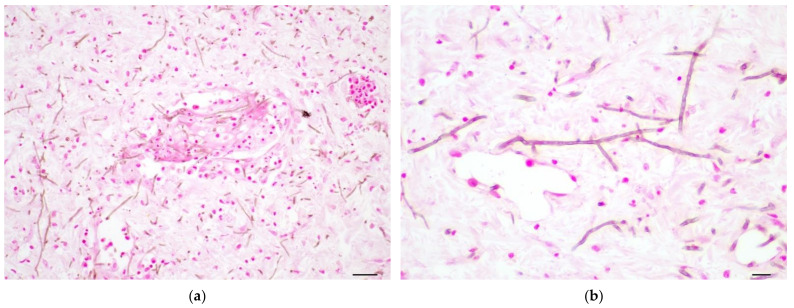
Fungal angioinvasion and histochemical staining of hyphae for melanin. (**a**) Melanized fungal hyphae are located in the lumen of a blood vessel and throughout the surrounding dermis accompanied by a dermal infiltrate of mononuclear cells. Fontana–Masson, bar = 20 µM. (**b**) High-magnification image of dermis from (**a**). Hyphae are slender, with parallel walls, transverse septa, and lateral branching; hyphae are stained brown using the Fontana–Masson technique indicative of the presence of melanin. Fontana–Masson, bar = 10 µM.

Fungal angioinvasion was represented by intertwined fungal hyphae present in the lumina of blood vessels of gill and viscera along with infiltrates of macrophages and lymphocytes, fibrin, cellular debris, and necrotic endothelium. Affected vessels were present throughout the integument, liver, kidney, gill arches, intestine, and eye (Figure 3a). Fungal hyphae were filamentous and slender, 2–3 µM in diameter, with parallel walls, septa, and lateral branching. Hyphae stained brown in routine hematoxylin and eosin-stained sections and were brown to black in sections prepared using the Fontana–Masson technique, consistent with the expected histochemical staining reaction of melanized fungi (Figure 3b).

Ciliated protozoa, consistent with scuticociliates, were identified on histologic examination of gill, skin, and eye in three individuals. Other histopathologic findings included myxozoan parasites consistent with *Myxobolus albi* in three individuals and ovarian degeneration in one individual.

Postmortem aerobic culture was recorded in a single case. Aerobic culture of the ovary was negative for bacterial growth in one individual (T12069).

### 3.4. Fungus Identification and Antifungal Drug Susceptibility Testing

**Figure A1:**
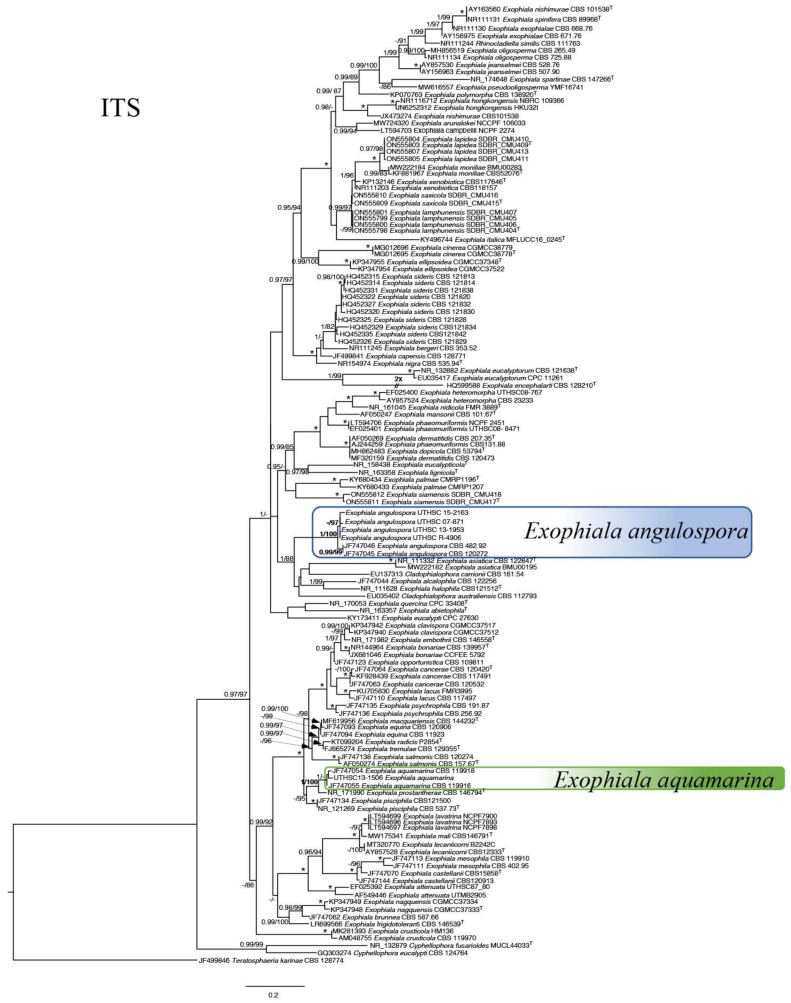
Maximum likelihood tree based on ITS showing the relationship of the isolates 9404 (UTHSC 15-2163); T12068 (UTHSC 13-1953), 3152 (UTHSC 07-871), and T12069 (UTHSC 13-1506) with representative *Exophiala* species and other species in the Herpotrichiellaceae. Numbers on the nodes indicate support values BI ≥ 0.95/BS ≥ 80%. * denotes BI = 1.00/BS = 100%. ^T^ = type species.

Sequences of isolates 9404 (UTHSC 15-2163), T12068 (UTHSC 13-1953), and 3152 (UTHSC 07-871) when subjected to an FTL local BLAST search in an updated database of ITS barcode identifiers for herpotrichiellaceous species gave similarity matches of 97-99% with *E. angulospora* CBS 482.92^T^. The top match for T12069 (UTHSC 13-1506) barcode BLAST was *E. aquamarina* CBS 119918 at 100% identity. A maximum likelihood phylogeny of the ITS using the most appropriate substitution model, TIM2 + F + I + G4, and rooted with *Teratosphaeria karinae* CBS 128774 and Bayesian inference were performed to confirm the presumptive species identification based on morphology and barcode. 9404, T12068, and 3152 were confirmed as *E. angulospora* with high support (BI = 1.00/BS = 100%) and *E. aquamarina* for UTHSC 13-1506 (BI = 1.00/BS = 100%) (Figure A1). A fourth postmortem culture sent to the FTL (3189 = UTHSC 07-1926) was identified as *Exophiala angulaspora* based on phenotypic characteristics alone.

Isolates identified as *E. angulospora* grew small restricted velvety black colonies on PFA. Tape and slide culture mounts showed angular conidia borne on phialides and sometimes annellides. Isolate T12069 (UTHSC 13-1506), identified as *E. aquamarina*, grew small restricted moist to velvety black colonies on PFA. Tape and slide culture mounts showed yeast-like cells and ellipsoidal conidia borne on phialides and annellides on terminal or intercalary conidiophores. Growth was observed for both species at 30 °C (86 °F) but not at 37 and 40 °C (98.6 and 104 °F).

Although there are no existing interpretive criteria for antifungal susceptibilities against these species, susceptibility testing of the isolates demonstrated good in vitro activity for itraconazole, voriconazole, and amphotericin B. The isolates were consistently resistant to fluconazole with MIC values of 32 μg/mL or higher (Table 3). Variability was observed with terbinafine, as the three *E. angulospora* isolates had MIC values of 2 μg/mL (the highest concentration tested), while the single *E. aquamarina* isolate had a lower terbinafine MIC (0.03 μg/mL).

### 3.5. PCR Detection of Exophiala from Formalin-Fixed Paraffin-Embedded Tissues

DNA extracts from FFPE tissue samples of eight lumpfish were tested using the lumpfish COX1 PCR assay. Six of eight lumpfish had FFPE samples that yielded COX1 sequences (205 bp after primer removal) which were identical to each other and to numerous lumpfish COX1 sequences in GenBank (data not shown). Of those COX1-positive DNA extracts, four tested positive using the *Exophiala* ITS1 PCR assay (Table 1) Two PCR-positive DNA extracts (T12069, T12068) yielded identical ITS sequences (143 bp after primer removal) to each other and to *E. aquamarina* ITS sequences originating from the tissues of: leafy seadragons (*Phycodurus eques*), weedy seadragons (*Phyllopteryx taeniolatus*), winter flounder (*Pseudopleuronectes americanus*), little tunnyfish (*Euthynnus alletteratus*) (GenBank Acc. Nos. JF747054-JF747061), fine flounder (*Paralichthys adspersus*) (MH813288), and sharphead flyingfish (*Hirundichthys oxycephalus*) (LC547432). The other two PCR-positive DNA extracts (6192, 3197) yielded identical ITS sequences (131 bp after primer removal and trimming) to each other and to a *Cyphellophora* sp. ITS sequence originating from a case of disseminated phaeohyphomycosis in lumpfish from Ireland (MG878986).

## 4. Discussion

Previous descriptions of phaeohyphomycosis in lumpfish have focused on postmortem sampling and pathologic description, with limited antemortem sampling or treatment attempts [6,7,31,32]. Aquarium-housed lumpfish provide a sample population of closely monitored individuals that allow fish health professionals to observe the course of disease and to attempt treatment on a small scale using medications that could be further studied for their use in aquaculture. Additionally, early identification via antemortem diagnosis in aquaculture can be used to rapidly identify infected individuals.

For fish with skin lesions, antemortem direct skin cytology for fungal elements resulted in presumptive diagnosis of fungal dermatitis in 10 cases in this retrospective study. This is an inexpensive and rapid diagnostic test in fish. External lesions are more likely to be noticed in the early stages of disease and can be easily screened for etiologic agents. The use of in-house cytology can inform further antemortem diagnostics, including biopsy and culture, allowing for molecular identification and fungal MIC testing.

Fungal cultures were performed on swabs of cutaneous lesions, on tissue biopsies, or in one instance on an aspirate of a skin cyst. Although this is a small sample size, all three fungal cultures from tissue biopsies were positive and allowed for definitive fungal identification. Of the eight fungal cultures performed on swabs of lesions, on the other hand, only four resulted in fungal growth. It is not clear if the negative cultures obtained were a result of sampling technique or selection bias by collecting biopsies from more advanced fungal lesions. 

Histopathologic findings in aquarium-housed lumpfish with *Exophiala* sp. infections were comparable to lesions previously described in cultured lumpfish [6,7,31,32] and in other aquarium-housed fish [8]. Visceral lesions were found in a total of 12/15 individuals (80%). In the twelve cases where phaeohyphomycotic lesions were documented in the skin or gills via histopathology, nine lumpfish (75%) had visceral lesions. In light of this high percentage of visceral involvement, future treatment approaches should address systemic infection and not be limited to topical treatments for external lesions. 

In cases without obvious skin lesions, the most common signs were non-specific signs of illness. These lumpfish had subjectively shorter survival times from the onset of clinical signs and were more often found dead rather than euthanized. Although antemortem diagnosis of *Exophiala* sp. lesions of the coelomic viscera has been assessed via ultrasound [52], this was not pursued in these cases. Three individuals had visceral lesions without histologic confirmation of fungal elements in the skin, although antemortem skin erosions were present in two of these lumpfish and skin cytology showed fungal elements. Since gross necropsy and sample collection were performed by the attending clinician, it is possible that representative samples of the integumentary lesions were not collected for histopathology. In addition to fungal elements, scutiociliates were reported on direct cytology in six cases. In four of these cases, these scutiociliates were the only findings on initial skin scrapes and preceded the presence of fungal elements in later lesions. Scuticociliates may be a source of skin trauma and provide a portal of entry for *Exophiala* infection [7,10,23]. PCR testing of formalin-fixed paraffin-embedded (FFPE) tissues from necropsy samples yielded positive results in four of eight lumpfish. *E. aquamarina* was identified in FFPE tissue samples from two lumpfish from the same institution. For one of those, *E. angulospora* was identified on antemortem fungal culture, and this culture was used as a positive control for the *E. angulospora* primer utilized in FFPE testing. Significant sequence disparity exists between the genetic targets for *E. angulospora* and *E. aquamarina* in this assay to discriminate between the two by amplicon sequence analysis. The discrepancy between culture and PCR results in this one lumpfish may indicate co-infection, infection at two different sampling sites, or a temporal shift in infection between species of *Exophiala*. It is also possible that culture growth of *E. angulospora* was preferential to that of *E. aquamarina*.

*Cyphellophora* sp. was identified from FFPE in two individuals where antemortem culture did not yield a fungal colony for molecular testing. The sequence of this *Cyphellophora* sp. was identical to a *Cyphellophora* sp. isolated from a lumpfish in Ireland and represents an additional causative agent of phaeohyphomycosis in lumpfish [32]. Due to its slow growth rate, *Cyphellophora* sp. is difficult to culture and may require selective media or procedures for isolation [53].

None of the attempted treatments in this retrospective study were considered effective as infection persisted in treated individuals. This is consistent with other reports of *Exophiala* spp. infection in *C. lumpus* and other fish [7,10,32]. Despite the reported in vitro susceptibility of some *Exophiala* isolates to antifungal drugs [28], there are no reported cases of successful treatment of fungal lesions in *C. lumpus.* Hyatt described successful treatment of cutaneous *E. aquamarina* in two valentine pufferfish (*Canthigaster valentini*) using Mohs’ Paste, followed by a combination of misoprostol, phenytoin, and enrofloxacin in a bioadhesive powder [54]. This protocol was not attempted in any of the fish in the current study; however, due to the apparent high percentage of visceral involvement in *C. lumpus*, it is unlikely for topical treatment alone to eliminate *Exophiala* infections in this species.

In humans, successful treatment of *E. dermatitidis* has been reported with long-term itraconazole or voriconazole and with a combination of surgical resection and medical therapy including amphotericin B, flucytosine, and ketoconazole [55,56]. In vitro activity of voriconazole, posaconazole, and itraconazole has been demonstrated in *E. dermatitidis* isolates from human infections, although amphotericin B and terbinafine may provide synergistic effects for treatment of refractory cases or severe infections [57]. The MIC data in the present study indicate that fluconazole treatment is likely to be ineffective for lumpfish. However, relatively low MICs were seen for amphotericin B, itraconazole, terbinafine, voriconazole, and miconazole. These drugs are candidates for systemic pharmacokinetic and pharmacodynamic studies in aquarium-housed lumpfish to determine whether effective plasma and tissue concentrations might be achievable.

Immune suppression is a significant risk factor for opportunistic fungal infections, including *Exophiala* spp. infections in humans [55,58]. Immune suppression can come from a variety of sources in aquarium and cultured fish, including but not limited to transport, conspecific aggression or trauma, or environmental stress. It is unclear whether individual fish that are infected with *Exophiala* spp. pose a risk to conspecifics in the same enclosure. While an infected individual could be shedding organisms into the environment, or transferring organisms through direct contact with other fish, the relative importance of these forms of transmission is not known in comparison to the route of general environmental exposure for these ubiquitous fungi. As such, decisions regarding isolation or culling of infected individuals, disinfection of tank systems, and management of exposed, healthy, conspecific fish remain at the discretion of the attending fish health professionals.

Of 15 individuals in this study, fungal identification to a species level was confirmed in 5 individuals via antemortem culture, with species identification in two additional individuals using PCR on postmortem FFPE samples. Obtaining a culture isolate provides total genomic DNA of greater integrity in support of DNA sequencing for molecular characterization and provides a viable specimen for clinically relevant, growth-dependent, in vitro testing, such as the broth microdilution method of antifungal drug susceptibility testing. Fungal cultures were not pursued in every case possibly due to financial or clinical concerns or because fungal cultures were pursued in one individual within a group, and the results were used to inform clinical decisions for cohorts. Fungal cultures should be considered for *C. lumpus* with ulcerative skin lesions, and results should be correlated to histopathologic findings.

There are several limitations of our retrospective study. Cases were selected from postmortem findings provided in pathology reports. As a result, full sets of antemortem diagnostics and husbandry parameters were not available in all cases. Inconsistent data between medical records from all institutions limited comparisons on the time that the fish had been in the collection and the source of individual lumpfish. Samples from each aquarium were subsets of the populations of lumpfish at these institutions. As a result, no conclusions can be made about the prevalence of phaeohyphomycosis in aquarium-raised lumpfish.

## 5. Conclusions

Melanized fungi represent an important disease concern in aquaculture, and for management of lumpfish as a species, both in production farming and in public aquaria. *Exophiala* spp. are a common cause of morbidity and mortality in aquarium-raised as well as cultured lumpfish. To date, there are no effective therapies for *Exophiala* infections in lumpfish. Black yeasts and close relatives belonging to the family Herpotrichiellaceae are difficult to distinguish by conventional methods because of their similar morphologies. The use of DNA sequencing of the ITS region and the introduction of a barcode identifier for herpotrichiellaceous species can provide a practicable tool in the identification of these species and diagnosis of infections [44]. Efficient antemortem diagnosis is possible using skin cytology and culture. Management of individuals with *Exophiala* infections in public aquaria may provide valuable information on the progression and possible treatment of *Exophiala* infections in lumpfish under all conditions.

## Figures and Tables

**Table 1:** Summary of cutaneous lesions and diagnostic testing in 15 lumpfish. Samples are listed in chronological order by date of postmortem examination *.

Institution	House ID	Cutaneous Lesions Present?	Direct Cytology Performed?	Fungal Elements on Skin Scrape	Biopsy	Antemortem Fungal culture	Culture Source	Culture Result	Isolate Identification by FTL on Antemortem Culture	Isolate Identification on Postmortem FFPE	MIC Data Available
NEAq	3152	Y	Y	Y	N	Y	Caudal tail lesion, Swab	Pigmented mold growth	*Exophiala angulospora*	None	Y
					N	Y	Dorsal skin lesion, Swab	Pigmented mold growth	*Exophiala angulospora*	None	Y
NEAq	3189	Y	Y	Y	N	Y	Skin lesion, Swab	Pigmented mold growth	*Exophiala angulospora* **	ND	Y
NEAq	3197	Y	Y	N	N	N				Closest match to *Cyphellophora guyanensis*	
Quebec	09N032	N	N	N/A	N	N				ND	
Quebec	09N038	N	N	N/A	N	N				ND	
Quebec	09N051	Y	N	N/A	N	N				ND	
NEAq	6192	Y	Y	Y	Y (not used for culture)	Y	Skin lesion, Swab	No growth		Closest match to *Cyphellophora guyanensis*	
NEAq	6193	Y	Y	Y	N	Y	Aspirate from Skin Cyst, Swab	No growth		ND	
					N	Y	Skin lesion, Swab	Pigmented mold growth		N/A	N
Quebec	11N210	Y	Y	Y	N	Y	Skin Lesion, Swab	No growth		ND	
Aq of the Bay	11028	Y	Y	Y	N	N				N/A	
WHSA	1103	Y	Y	Y	N	N				None	
Quebec	12N091	N	N	N/A	N	N				ND	
NADC	T12069	Y	Y	Y	Y	Y	Skin Lesion, Swab	No growth		ND	
						Y	Skin lesion, Biopsy	Pigmented mold growth	*Exophiala aquamarina*	Identical to *Exophiala aquamarina*	Y
NADC	T12068	Y	Y	Y	Y	Y	Skin lesion, Swab	No growth, bacterial contamination		ND	
						Y	Skin lesion, Biopsy	Pigmented mold growth	*Exophiala angulospora*	Identical to *Exophiala aquamarina*	N
NEAq	9404	Y	Y	Y	Y	Y	Skin lesion, Biopsy	Pigmented mold growth	*Exophiala angulospora*	ND	N

FTL = Fungus Testing Laboratory, Department of Pathology and Laboratory Medicine, Long School of Medicine, University of Texas Health San Antonio, Texas USA, FFPE = Formalin-fixed paraffin-embedded tissue samples, NEAq = New England Aquarium, Quebec = Aquarium du Québec, Aq of the Bay = Aquarium of the Bay, WHSA = Woods Hole Science Aquarium, NADC = National Aquarium, Washington DC, ND = not done, N/A = lumpfish COX1 PCR negative, not tested by FFPE fungal PCR, none = negative FFPE fungal PCR result. * Two fungal cultures were obtained for individuals 3152, 6193, T12069, and T12068 over the course of their clinical disease. ** = Identified by phenotypic characteristics only.

**Table 2:** Distribution of phaeohyphomycotic lesions identified on histopathology by anatomic location from 15 lumpfish. Samples are listed in chronological order by date of postmortem examination.

Institution	House ID	Integument/Muscle	Gill	Kidney (Cranial and/or Caudal)	Liver	Heart	Spleen	Intestine	Coelomic Cavity/Serosa	Gonad	Stomach	Eye	Mesentery
NEAq	3152	X	X	X	X	X						X	
NEAq	3189	X											
NEAq	3197	X	X		X		X	X		X	X		
Quebec	09N032			X	X		X						
Quebec	09N038	X	X	X			X	X					
Quebec	09N051		X	X	X				X				
NEAq	6192	X	X	X	X	X	X						
NEAq	6193				X	X							
Quebec	11N210								X				
Aq of the Bay	11028	X	X	X									
WHSA	1103	X											
Quebec	12N091	X	X	X	X	X	X	X	X				
NADC	T12069	X											
NADC	T12068	X	X	X		X							
NEAq	9404	X	X	X		X	X	X		X	X		X
	Total:	11	9	9	7	6	6	4	3	2	2	1	1

**Table 3:** In vitro susceptibilities of the four *Exophiala* isolates obtained from skin lesions (two isolates from the same lumpfish) against five antifungal agents. MIC values are expressed in μg/mL. AMB = Amphotericin B, FLC = Fluconazole, ITC = Itraconazole, VRC = Voriconazole, TRB = Terbinafine, MON = Miconazole.

Institution	House ID	Species	Location	AMB	FLC	ITC	VRC	TRB	MON
NEAq	3152	*E. angulospora*	Caudal tail	0.25	32–64	0.125	0.5	2	~
		*E. angulospora*	Dorsum	0.25	32	0.06	0.5	2	~
NEAq	3189	*E. angulospora*	Skin lesion	2	32–64	0.25	0.5	2	~
NADC	T12069	*E. aquamarina*	Skin lesion	~	~	0.5	~	0.03	0.25

## Data Availability

Not applicable.

## Appendix

**Table A1:** PCR primer pairs targeting the fungal internal transcribed spacer 1 (ITS1) region and the lumpfish cytochrome oxidase 1 (COX1) gene.

Primer Name	Primer Sequence (5′-3′)	Target	Amplicon Size (bp)
Exophiala-F1	ACCYCTTGTTGCTTCGGC	ITS1	181
Exophiala-R1	GCCAGAACCAAGAGATCCGT		
Lumpfish-F1	AATCATAATCGGGGGCTTTG	COX1	245
Lumpfish-R1	CCGCGAGGTGTAAAGAAAAG		
